# A case report of atypical human infection with *Streptococcus suis* purulent meningitis

**DOI:** 10.1097/MD.0000000000047518

**Published:** 2026-01-30

**Authors:** Ailing Han, Jialing Guo, Yidan Wang, Huiling Han

**Affiliations:** a The Hospital of Chifeng, Chifeng, China; b The Seventh Medical Center of the Chinese PLA General Hospital, Beijing, China; c Inner Mongolia Hospital of Xuan Wu Hospital Capital Medical University, Chifeng, China.

**Keywords:** diagnosis, prevention and treatment, purulent meningitis, *S suis* infection

## Abstract

**Rationale:**

We report a case of purulent meningitis caused by *Streptococcus suis* infection in a 65-year-old male patient. The patient had no typical epidemiological history and was admitted with fever, headache, and psycho-behavioral abnormalities. Compared with previous reports, this case has particularities in terms of diagnostic methods, epidemiology and clinical manifestations. Combined with the literature analysis, it aims to provide reference for clinical development of targeted diagnosis and treatment plan, clear research direction and guide public health institutions to carry out traceability prevention and control.

**Patient concerns:**

Fever, headache, psychiatric behavioral abnormalities (delirium, confusion), neck stiffness, and a positive Kernig’s sign, indicating meningeal irritation; there were no symptoms such as nausea, vomiting, or convulsions.

**Diagnoses:**

Purulent meningitis caused by *S suis* infection.

**Interventions:**

During the initial stage, broad-spectrum antibiotics such as meropenem and vancomycin were administered empirically. After confirmation of *S suis* infection, the treatment was adjusted to a high-dose penicillin combined with vancomycin. Supportive therapies, including neurotrophic agents, hepatoprotective drugs, and electrolyte imbalance correction, have also been provided.

**Outcomes:**

Following treatment, the frequency of fever decreased, consciousness was restored, and inflammatory marker levels improved. Hearing loss remained a sequela. The patient was discharged approximately 2 months after hospitalization and continued penicillin therapy, with instructions to return for follow-up if any discomfort occurred.

**Lessons:**

This case suggests that vigilance is required even in the absence of a typical epidemiological history; attention should be paid to including next generation sequencing technology in the diagnostic process of unexplained meningitis in order to shorten the time of diagnosis, guide precise anti-infective treatment, avoid antibiotic abuse and reduce drug resistance. When the clinical manifestations are atypical, the influence of pathogenic variants or early treatment needs to be considered and should not be restricted to classical manifestations. In addition, reporting such cases is of great public health importance to regional healthcare facilities and helps raise awareness of zoonoses diagnosis, even in non-endemic areas.

## 1. Introduction

*Streptococcus suis* is an emerging zoonotic pathogen that was first isolated from pigs in 1963 and later confirmed to be transmitted from pigs to humans mainly through close contact with sick pigs.^[1,2]^ In 1968, Danish scholars first reported human infections with *S suis*,^[3]^ followed by reports from North America to South America, Europe, Asia, Australia, and New Zealand. An outbreak occurred in Jiangsu Province in 1998 and Sichuan Province in 2004.^[4,5]^ The outbreak in Jiangsu Province was the first large-scale epidemic of human infection with *S suis* in China. A total of 25 cases have been reported, of which 14 died, and the mortality rate was as high as 56%. This outbreak was dangerous, particularly in the case of toxic shock syndrome. The outbreak in Sichuan Province is the largest and most serious epidemic of *S suis* infection in humans worldwide, with a cumulative number of 215 reported cases and 39 deaths, with a mortality rate of 18.1%. In recent years, sporadic cases or small clustered epidemics have also been reported in other provinces and cities in China (e.g., Guangdong, Guangxi, Hunan, Jiangxi, etc).^[4,5]^

*S suis* is a zoonosis caused by infection with various pathogenic strains of *S suis*. The main symptoms are fever, chills, headache, and loss of appetite, which are characterized by insidious onset, rapid progression, critical condition, and high mortality.^[6,7]^ *S suis* can colonize the upper respiratory tract of pigs without causing disease, however, invasive streptococci can cause serious clinical conditions, such as meningitis, arthritis, and sepsis, and even lead to acute death.^[8,9]^ Among them, purulent meningitis is the most common type of infection, accounting for approximately 50% to 70% of reported cases, and typical symptoms include fever, headache, neck stiffness, disturbance of consciousness, and psycho-behavioral abnormalities.^[8,9]^ Approximately 20% to 30% of patients present with sepsis, often with high fever, chills, leukocytosis, significantly elevated C-reactive protein (CRP) and procalcitonin (PCT) levels, and severe cases can develop septic shock. Clinical manifestations such as endocarditis and arthritis are extremely rare, and most cases are due to the appearance of migratory lesions in sepsis/bacteremia. Endocarditis is more common in patients with an underlying heart valve disease. A very small proportion of patients develop Streptococcal Toxic Shock Syndrome (STSS) (<5%), but the mortality rate is extremely high and long manifests as rapidly progressive multiple organ failure, hypotension, Disseminated Intravascular Coagulation (DIC), etc.^[8,10,11]^ World Organization for Animal Health (OIE) has classified swine streptococcal disease as a category B epidemic disease, while China has classified it as a category II animal epidemic disease.

In this paper, we report a case of purulent meningitis due to atypical *S suis* infection in the Chifeng area admitted to Chifeng Hospital of Inner Mongolia, and conduct a retrospective analysis in conjunction with relevant literature.

## 2. Case presentation

### 2.1. Clinical presentation

A 65-year-old male farmer, was admitted to our hospital on September 12, 2022. Three days before admission, the patient developed fever, headache, mental and behavioral abnormalities without obvious inducement, and body temperature was not measured. After self-administration of antipyretic drugs, the fever was relieved, and the patient babbled, and could not perform daily activities normally. He was transferred to the emergency department of our hospital after undergoing cranial computed tomograph (CT) and other examinations at a local hospital. Perfected head magnetic resonance imaging (MRI) and head CT in the emergency department showed “multiple cerebral infarctions and brain atrophy.” Lumbar puncture cerebrospinal fluid pressure was 400 mm H_2_O; cerebrospinal fluid routine:WBC 1.155 × 10^9^/L, yellowish color; cerebrospinal fluid biochemistry: trace total protein 3169 mg/L, glucose <0.00 mmol/L, chlorine 118.6 mmol/L. The patient was admitted to the neurology department of our hospital due to “purulent meningitis.” During the course of the disease, there was no nausea, vomiting, limb twitching, limb immobility, poor diet and sleep, normal urine and defecation, or change in body weight. History of hypertension for 10 years on chronic medication. He had a history of heavy drinking for more than 20 years. He denied any history of diabetes or heart disease, had no long-term history of living in other places, and had no history of living in epidemic areas. Physical examination revealed a body temperature of 37.6°C, pulse of 71 beats/min, respiration 18 breaths/min, blood pressure 151/108 mm Hg, confusion, babbling, uncooperative physical examination, bilateral pupils of equal size and round, approximately 3 mm in diameter, sensitive light reflex, uncooperative tongue extension, no significant abnormalities in limb muscle strength or muscle tone, uncooperative synkinesis examination, uncooperative deep and superficial sensory examination, ciliary reflex (++), pathological signs (−), strong neck positive 4 transverse fingers, and positive Klinefelter sign. Wada drinking water test grade I. Premorbid modified rankin scale 0, low risk of venous thromboembolism, Glasgow coma scale (GCS) 13.

Epidemiological history: The patient was born in Balinzuo Banner, Chifeng City, Inner Mongolia, and had no long-term history of residence in other places or contact with epidemic areas. The sanitary environment around the family and residence was good, and he denied that there were pigs, poultry, livestock farms, and slaughter houses in and near him. He denied drinking undercooked milk or goat milk, drinking raw water, a history of long-term alcohol consumption, and no macroscopically visible skin damage to the body. Consumption of the large intestine from sick pigs 1 week before the onset of illness (the patient reported being cooked) was suspected to be exposured in this case. Close contacts with the patient were his wife and children, both of whom had no disease.

The patient was admitted to the neurology department of our hospital with purulent meningitis. Further tests and examinations, including routine blood and urine tests and, chest radiography, were performed to monitor the patient’s condition.

### 2.2. Interventions

On September 14, the patient had a severe infection, and lumbar puncture was performed to identify changes in the cerebrospinal fluid. On September 15, according to the consultation opinion of the Department of Infectious Diseases, Meropenem combined with vancomycin was added for treatment, and the current anti-inflammatory treatment regimen was continued to monitor the changes in relevant test indicators, on September 18, bacterial culture of cerebrospinal fluid showed no bacterial growth for 2 days, and microscopic examination of cryptococcal smear was negative. On September 20, no growth of bacteria or anaerobic bacteria was found in the blood culture for 5 days. The patient’s symptoms were relieved slightly. Current symptomatic and supportive treatments, such as anti-inflammation, trophic nerve, liver protection, and correction of ion disturbance were continued. Cerebrospinal fluid test was performed electively to complete cranial MRI reexamination. On September 24, cerebrospinal fluid smear microscopy showed negative cryptococcal smear microscopy, cerebrospinal fluid bacterial smear microscopy showed negative, cerebrospinal fluid smear acid-fast staining microscopy showed negative, the patient still had intermittent fever, and further perfected: fungal direct smear microscopy (throat swab), G test, GM test, MN test, after the results returned, the current treatment regimen was continued, and antibiotic treatment was adjusted according to the condition. On September 27, fungal smear microscopy showed no hyphae or spores, and the patient still had intermittent fever. Infectious disease consultation: Rocephin was recommended for continued treatment, on October 5, microscopic examination of the urine fungal smear showed no hyphae or spores, and monitoring was continued. The patient’s condition remained stable, but fever symptoms rebounded further after adjusting ceftriaxone. Infectious disease consultation: We decided to adjust antibiotics to classical treatment with vancomycin and meropenem, and further send cerebrospinal fluid for second-generation gene sequencing to identify pathogenic bacteria. Lumbar puncture was reexamined to observe changes in the condition. On 10.8 days, acid-fast staining microscopy of the smear was negative, fungal smear microscopy showed no hyphae or spores, cryptococcal smear microscopy was negative, bacterial smear microscopy was negative, and cerebrospinal fluid culture showed no bacteria for 2 days. On October 13, the patient’s condition was stable, the current treatment regimen was continued, and adjuvant therapy with Qingre Bawei Capsules was added, after which the patient’s fever was not significantly improved. Consider adding C-ball to modulate immunotherapy. On October 15, the second-generation gene sequencing results returned, suggesting *S suis* positive. After communicating with the patient’s family, the patient switched to penicillin 4.8 million units/6 h ivgtt for anti-inflammatory treatment, and changes in the condition were observed. On October 20, the frequency of fever and maximum body temperature decreased than before, the patient had clear consciousness, normal diet and spirit, and the current anti-inflammatory treatment regimen was penicillin combined with vancomycin. On October 26, the frequency of fever decreased, the highest body temperature decreased significantly, anti-inflammatory treatment regimen penicillin combined with vancomycin, albumin, red blood cells were higher than before, considering that the nutritional status was gradually improving, continue the current treatment regimen. On November 1, the patient had PCT 0.035 ng/mL, CRP 24.5 mg/L, white blood cell 3.98 × 10^9^/L, and inflammatory indicators decreased than before, suggesting that inflammation was controlled. On November 8, the patient’s condition was stable and further improved, with normal white blood cells. At present, the total course of vancomycin application has been completed in 6 weeks. The drug was discontinued, and the patient was given single agent penicillin for anti-inflammatory treatment. On November 14, the patient had no fever, clear consciousness, hearing loss, normal spirit, Wada drinking water test grade I, GCS 15 points, discharge, medication guidance; Penicillin 4.8 million units/time/6 h ivgtt, continued for 2 weeks; came to the hospital 2 weeks after discharge for routine reexamination of blood, liver and kidney function, blood lipid and blood glucose, coagulation 4 items, head MRI, lumbar puncture cerebrospinal fluid examination, and so on.

### 2.3. Assistant examination

#### 2.3.1. Peripheral blood routine

From September 11, 2022, to November 14, 2022, a total of 8 peripheral blood routine examinations were conducted. The results indicated a significant increase in WBC count and NEUT% at the initial stage of admission, providing crucial laboratory evidence for the diagnosis of purulent meningitis. The initial decrease in PLT and extremely low LY% suggest severe infection and a strong stress state in the body. The continuous decline in WBC and NEUT% and the rebound of PLT were key dynamic indicators of effective antibiotic treatment. The leukopenia and anemia observed during the treatment process were either due to the disease itself or possible side effects of drug therapy. The gradual normalization of various routine blood indicators in the later stage indicated successful recovery from acute severe infection (Table 1).

**Table 1:** Routine peripheral blood test results.

	WBC (10^9^/L)	NEUT (%)	LY (%)	HGB (g/L)	PLT (10^9^/L)
September 11, 2022	12.97	92.40	2.60	126.00	150.00
September 13, 2022	7.50	78.40	10.80	124.00	149.00
September 22, 2022	6.63	76.80	9.40	115.00	586.00
September 30, 2022	2.87	56.10	30.00	116.00	233.00
October 4, 2022	2.20	63.10	25.90	109.00	221.00
October 13, 2022	2.45	45.70	34.30	102.00	235.00
October 24, 2022	3.50	55.70	18.60	84.00	301.00
November 14, 2022	4.18	66.20	15.60	90.00	351.00

WBC = white blood cell, NEUT (%) = neutrophil percentage, LY (%) = lymphocyte percentage, HGB = hemoglobin, PLT = platelet.

#### 2.3.2. Inflammatory markers

**Figure 1.**
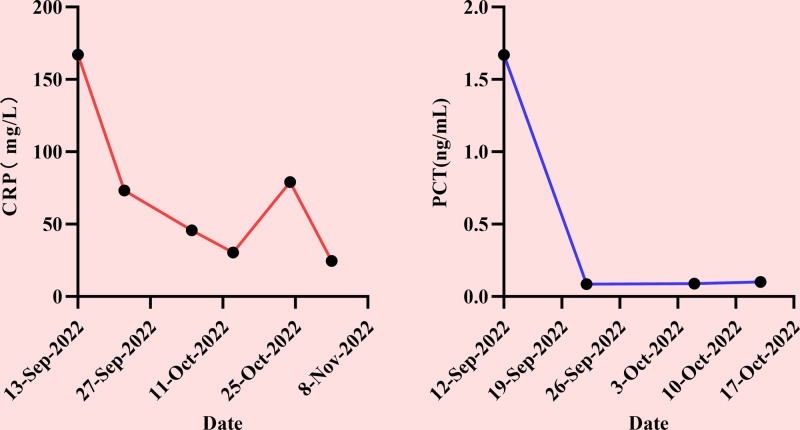
Change trend of inflammatory factors CRP and PCT. CRP = C-reactive protein, PCT = serum procalcitonin.

On admission, CRP and PCT levels peaked, indicating severe bacterial infection and, supporting the initial diagnosis of “bacterial meningitis.” During treatment, the 2 values showed an overall decreasing trend, suggesting that the treatment was effective, and the inflammatory response in the body gradually decreased. CRP levels rebounded during treatment, suggesting complex conditions or complications, which also explains why physicians continuously adjusted treatment regimens and actively sought etiological diagnosis during treatment. Before discharge, PCT levels decreased to the normal range and CRP levels decreased significantly, indicating that bacterial infection with systemic inflammation was largely controlled (Fig. 1).

#### 2.3.3. Routine and biochemical indicators of cerebrospinal fluid

Cerebrospinal fluid white blood cell (WBC) values and mononuclear cell ratios showed a decreasing trend, cerebrospinal fluid red blood cell values showed an increasing trend, and no multinucleated cells (SCE-FY) were observed (Table 2). The biochemical indices of cerebrospinal fluid showed that the index of trace total protein increased significantly at the early stage of hospitalization, reaching 3169 mg/L. With further treatment, the value of trace total protein showed a downward trend. Cerebrospinal fluid glucose and chloride measurements were lower than normal values, and cerebrospinal fluid glucose measurements were <0.00 mmol/L on September 11 (Table 3).

**Table 2:** The results of CSF routine.

	YS	TMD	NGX	WBC (10^6^/L)	RBC	WBC-NO% (%)	SCE-FY (%)
September 11, 2022	Yellowish	Micromixture	No clot	1155	20	80	Not seen
September 14, 2022	Yellowish	Micromixture	No clot	611	43	57	Not seen
September 22, 2022	Yellowish	Clear and transparent	No clot	56	78	22	Not seen
October 5, 2022	Colourless	Clear and transparent	No clot	19	95	5	Not seen
November 24, 2022	Colourless	Clear and transparent	No clot	15	Not tested	Not tested	Not seen

CSF = cerebrospinal fluid, NGX = coagulability, RBC = red blood cell of cerebrospinal fluid, SCE-FY = multinucleated cell ratio, TMD = transparency of cerebrospinal fluid, YS = color of cerebrospinal fluid, WBC = white blood cell of cerebrospinal fluid, WBC-NO% = mononuclear cell ratio.

**Table 3:** The results of CSF biochemistry.

	CL (mmol/L)	GLU (mmol/L)	mTP (mg/L)
September 11, 2022	118.6	<0.00	3169
September 14, 2022	117.9	0.61	2418
September 22, 2022	119.4	2.39	1285
October 5, 2022	119.4	1.81	712
November 24, 2022	124.9	2.09	935

CSF = cerebrospinal fluid, CL = chloride, GLU = cerebrospinal fluid glucose, mTP = micrototal protein.

Cerebrospinal fluid glucose is significantly reduced (even undetectable), mainly due to 2 mechanisms: one is the massive consumption of bacteria and inflammatory cells, and the other is sugar transporter dysfunction, which is a hallmark feature of severe bacterial meningitis. Cerebrospinal fluid total protein levels are markedly elevated, which directly reflects severe disruption of the blood–brain barrier. The cerebrospinal fluid pattern of “very low sugar and very high protein” is a direct pathophysiological embodiment of the multiplication of bacteria in the central nervous system, triggering a severe inflammatory response and seriously destroying the blood–brain barrier.

#### 2.3.4. Imaging findings

**Figure 2.**
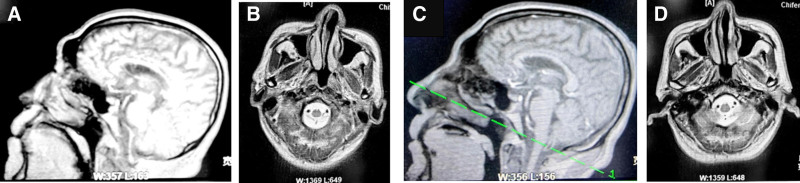
The result of MRI. (A, B) The MRI results of September 21: an abnormal signal is noted in the occipital horn of the right lateral ventricle, along with meningeal enhancement. Based on the medical history, the findings are suggestive of purulent meningitis accompanied by a small amount of fluid accumulation in the ventricles. (C, D) The MRI results of October 6: meningeal enhancement is noted in the right frontoparietal region. Based on the medical history, the findings are consistent with purulent meningitis. The conclusion is largely consistent with the findings from September 21.

Cranial MRI contrast-enhanced imaging showed abnormal signal intensity in the occipital horn of the right ventricle with meningeal enhancement and ventricular effusion. Combined with the medical history, the initial diagnosis of purulent meningitis with a small amount of intraventricular effusion was made (Fig. 2).

The MRI findings suggested the seriousness and complexity of the infection. Meningeal enhancement is a direct imaging marker of purulent meningitis and reflects vascular congestion, inflammatory cell infiltration, and blood–brain barrier disruption in the leptomeninges/arachnoid membrane after the pathogens break through the blood–brain barrier. Ventricular effusion requires high vigilance for the possibility of ventriculitis, pathogens can enter the ventricular system retrogradely along the cerebrospinal fluid circulation pathway, and intraventricular effusion is a manifestation of infection involving the ventricular lining. Ventriculitis is a complicated meningitis, meaning that infection is deeper, it is more difficult to penetrate antibiotics, it is more likely to lead to cerebrospinal fluid circulation disorders, and it is associated with a high risk of mortality and neurological sequelae. The patient presented with multiple cerebral infarcts on CT and the abnormal signal on MRI in the context of meningitis needs to be interpreted with caution. Bacterial infection can trigger a strong inflammatory response and vasculitis, leading to secondary, infectious vasculitic cerebral infarction, which is distinct from traditional atherosclerotic cerebral infarction in mechanism and treatment focus. Therefore, abnormal signals on MRI are likely not independent cerebrovascular events, but complications of severe central nervous system infection. In purulent meningitis, inflammatory exudates from the basal cisterns and outlet of the fourth ventricle mechanically obstruct the cerebrospinal fluid circulation pathway and cause acute obstructive hydrocephalus. Although the patient was not definitively diagnosed with hydrocephalus, ventricular effusion was an early manifestation of the condition.

These imaging findings were highly consistent with the patient’s severe neurological deficits (e.g., confusion, babbling, and neck stiffness) on admission. Ventricular system involvement and underlying parenchymal inflammation (abnormal signals) together explain their dramatic psycho-behavioral abnormalities and decreased level of consciousness, suggesting that inflammation causes widespread effects on the parenchymal and ventricular systems.

#### 2.3.5. Etiological examination

During hospitalization, 24 etiology-related examinations were performed, including cerebrospinal fluid, venous blood, sputum, throat swabs, urine, and feces. Specific examinations are shown (Table 4). The results were negative except for Candida glabra detected on stool fungal culture.

**Table 4:** The results of routine pathogen examination.

Specimen type	Item	Date (2022)	Results
Cerebrospinal fluid	General bacterial culture	9.14, 10.05	–
	Acid-fast staining microscopy of smear	9.14, 9.22, 10.05	–
	Direct smear Gram stain microscopy	9.22, 10.05	–
	Cryptococcus staining microscopy	9.14, 9.22, 10.05	–
Venous blood	Bacterial culture + identification of sterile body fluids	9.14, 10.08, 10.14	–
	Anaerobic culture + identification	9.14, 10.03, 10.08, 10.14	–
Sputum	Microscopic examination of fungal smear	10.05	–
	Smear acid-fast human chromoscopy	10.06	–
Throat swab	Microscopic examination of fungal smear	9.26, 10.04	–
Urine	Microscopic examination of fungal smear	10.04	–
	Fungal culture	11.01	–
Feces	Fungal culture	10.06	*Candida glabra*

On October 5, 2022, the cerebrospinal fluid was sent for next generation sequencing technology (NGS) to identify the pathogen, and the results reported on October 15 suggested that *S suis* was positive.

### 2.4. Outcome and follow-up

On November 14, 2022, the patient had no fever, clear consciousness, hearing loss, normal spirit, Wada drinking water test grade I, and a GCS score of 15. Discharge, medication guidance; Penicillin 4.8 million units/time/6 h ivgtt, continued for 2 weeks. He was asked to visit the hospital for reexamination at 1, 3, and 6 months after discharge, and his condition changed at any time in our department.

## 3. Discussion

### 3.1. Innovations in diagnostic techniques

One of the core implications of this case is to highlight the limitations of traditional etiological detection methods and the value of NGS in the diagnosis of difficult infectious diseases.

Patients underwent up to 24 microbiological examinations after admission, covering various methods such as smear and culture, and the results were negative. This dilemma of negative culture poses a serious clinical challenge. Bacterial culture is the gold standard for the diagnosis of bacterial meningitis,^[12]^ When it repeatedly presents negative results, doctors lose the most direct etiological evidence, and the diagnostic work regresses from “confirmation” to “speculation.” In the treatment process of this case, doctors have successively used potent broad-spectrum antibiotics such as meropenem, vancomycin, and ceftriaxone, covering the vast majority of common and resistant community-acquired and hospital-acquired bacteria, but the patient’s treatment response is poor, which clearly shows that even “top” empirical treatment may not be effective in controlling the infection in the case of unknown pathogens, resulting in prolonged course of the disease and increasing the risk of antibiotic-related side effects and drug-resistant bacteria. In this case, the cerebrospinal fluid showed atypical findings, multinucleated cells were not observed at all, it was easy to direct the direction of diagnosis to viral meningitis, tuberculous meningitis or fungal meningitis, and clinicians were forced to perform exclusive diagnosis, which took a lot of time, medical resources and patient patience. Cerebrospinal fluid NGS technology reflects its value when the above traditional diagnostic methods are in trouble. NGS directly extracts all nucleic acids from clinical samples for sequencing and is not affected by factors such as bacterial activity, antibiotic use before sampling, and difficulty in fastidious growth. It is able to detect all bacteria, viruses, fungi, and parasites that may be present in samples at one time. Which is particularly important for the diagnosis of relatively rare and easily ignored pathogens such as *S suis* infection in non-epidemic areas.

The present case suggests that negative culture no longer indicates the end point of diagnosis in the era of molecular diagnostics. Etiological diagnosis is the cornerstone for the treatment of infectious diseases. For purulent meningitis, especially in cases with negative multiple cultures and critical condition, antibiotics should not be discontinued or replaced blindly. Cerebrospinal fluid NGS should be used a first-line supplementary diagnostic tool as early as possible, which can significantly shorten the diagnostic cycle, achieve accurate treatment and improve the prognosis of patients.

### 3.2. Atypical clinical presentation challenges early diagnosis and treatment

Compared with previously reported cases of infection, the present case showed significant uniqueness and challenges in diagnostic methods, epidemiological history, and clinical features.

In previous clinical cognition, positive cerebrospinal fluid or blood culture is the “gold standard” for the diagnosis of meningitis caused by *S suis* infection.^[12]^ However, during hospitalization for more than a month in this case, all the results of blood culture, cerebrospinal fluid culture, and smear microscopy (including bacteria, fungi, and acid-fast bacilli) were negative, which made the diagnosis impasse and the pathogen could not be confirmed by conventional means. The final diagnosis relies solely on NGS of cerebrospinal fluid, which suggests that NGS technology plays a key role as a decisive diagnostic tool when traditional methods fail and that the load of pathogens in this case may be extremely low or have been partially inhibited by antibiotics, resulting in failure to capture by conventional methods.

In terms of epidemiological history, the route of exposure is vague and atypical. The vast majority of previous cases had a clear history of sick/dead pig contact, slaughter, or consumption of raw pork.^[13]^ The novelty of this case is that the only suspected exposure was to the “cooked” pig large intestine, and the patient denied direct contact with sick pigs, which is different from the typical percutaneous wound infection or the route of dealing with raw meat infection, suggesting that even if seemingly cooked food is consumed, there may still be a risk of infection if the treatment process is improper or the heating is incomplete, which broadens our understanding of the complexity of the transmission route of *S suis* and reminds public health authorities and clinicians that they should not only pay attention to whether they have direct contact when asking about the epidemiological history.

Clinical manifestations were atypical. In previous cases, multinucleated cells were usually predominant in the cerebrospinal fluid of *S suis* meningitis.^[14,15]^ In this case, multinucleated cells were not found in the cerebrospinal fluid routine, but mononuclear cells were predominant. This manifestation was more in line with the clinical features of viral meningitis or tuberculous meningitis, which can easily lead to misdiagnosis. In addition, there were significant psycho-behavioral abnormalities in this case. Although meningitis itself was suspected to cause psychiatric symptoms, this patient presented with babbling, psycho-behavioral abnormalities as as one of the prominent manifestations, even masking other typical symptoms at the early stage, which is also an atypical mode of onset that suggests the need for vigilance in clinical practice.

### 3.3. Enhancing clinical alertness to nontraditional epidemic areas

An outbreak of *S suis* infection occurred in Jiangsu Province in 1998 and Sichuan Province in 2004 in China.^[4,5]^ In recent years, sporadic cases or small clustered epidemic reports have been reported in other provinces and cities (such as Guangdong, Guangxi, Hunan, Jiangxi, etc), but the scale is very small. *S suis* meningitis is extremely rare in Chifeng City, Inner Mongolia, and there have been few reports of its occurrence. The successful diagnosis and treatment of this case are of great significance for improving the cognition and alertness of medical staff in nontraditional epidemic areas. This reminds physicians that *S suis* infection should be included in the differential diagnosis of meningitis patients with a history of exposure to pork products, even in areas where there is no mass pig industry. These cases also reveal possible regulatory loops in food processing and circulation. Strengthening education on pig quarantine and food safety and advocating thoroughly cooked meat are key to preventing such zoonoses.

## 4. Conclusion

Although public awareness of *S suis* infection has increased over the past few years, there remains a lack of research on pathogenesis and important issues in the development of cross-protective vaccines. However, there are no effective cross-protective commercially available vaccines, autovaccines, and antibiotics to control *S suis* disease, which is also a research focus that should be addressed by basic researchers and clinicians in the future.^[16]^

The diagnostic tortuosity of this case and the clinical complexity revealed highlight the current gaps in the field of human infection with *S suis* disease prevention and control. This suggests that future research should focus on the following key directions: Vaccine research and development: In view of the dual threat of *S suis* to the pig industry and public health, the development of effective cross-protective vaccines is fundamental way out for control. Future studies should focus on screening for conserved protective antigens and exploring immunization strategies suitable for humans versus pigs; Rapid diagnostic techniques: The application of bedside rapid detection techniques and NGS should be popularized and optimized to overcome the limitations of traditional culture and achieve early and accurate identification of infections, to guide timely targeted clinical treatment; Pathogenesis and immune mechanism: It is necessary to elucidate the molecular mechanism of *S suis* breaking through the host barrier, triggering an excessive inflammatory response and leading to nervous system injury in more depth, providing a target for the development of new intervention methods; Optimization of antibiotic regimens: In view of the increasing problem of antibiotic resistance worldwide, it is necessary to evaluate and optimize antibiotic regimens and explore new antimicrobial agents in larger clinical studies.

Only through sustained efforts across multiple disciplines can we effectively address the ongoing challenges posed by this ancient yet emerging zoonotic disease.

## Acknowledgments

The authors are very grateful to the patient for participating in this study.

## Author contributions

**Conceptualization:** Huiling Han.

**Data curation:** Ailing Han.

**Formal analysis:** Jialing Guo.

**Investigation:** Yidan Wang.

**Writing – original draft:** Ailing Han.

**Writing – review & editing:** Huiling Han.
